# Assessment of Individual and Combined Toxicities of Four Non-Essential Metals (As, Cd, Hg and Pb) in the Microtox Assay

**DOI:** 10.3390/ijerph2006030014

**Published:** 2006-03-31

**Authors:** Ali B. Ishaque, Linda Johnson, Tonya Gerald, Dwayne Boucaud, Joseph Okoh, Paul B. Tchounwou

**Affiliations:** 1 Department of Natural Sciences, University of Maryland Eastern Shore, Princess Anne, Maryland 21853; 2 Molecular Toxicology Research Laboratory, NIH-Center for Environmental Health, College of Science, Engineering and Technology, Jackson State University, Jackson, MS 39217, USA.

**Keywords:** Combined toxicities, Non-essential metals, Microtox assay

## Abstract

Although most researches with non-essential metals (NEMs) have been done with single or individual metals, in reality, organisms are often exposed to multiple contaminants at the same time through the air, food and water. In this study, we tested the toxicity of four NEMs, As, Cd, Pb, and Hg, individually and as a composite mixture using the microtox bioassay. This assay uses the reduction of bioluminescence of the bacterium *Vibrio fischeri* as a measure of toxicity. The concentrations of each chemical in the mixture were based on multiples of their maximum contaminant levels (MCLs) set by the U.S. EPA. The highest concentration of exposure was 20 times the MCL, which translated into 200, 100, 40 and 300 ppb for As, Cd, Hg and Pb, respectively. The ratio for the mixture from these concentrations was 10:5:2:15 for As, Cd, Hg and Pb, respectively. Among the individual metals tested, the ranking of toxicity was Hg>Pb>Cd>As based on the EC50 values of 109, 455, 508 and 768 ppb for Hg, Pb, Cd and As, respectively. The EC50 for the composite mixture was 495% MCL which translated into nominal concentrations of 49, 25, 10 and 74 ppb for As, Cd, Hg, and Pb, respectively. Overall, the EC50 value of each NEM within the mixture was lower than the EC50 of the individual chemical; an evidence of synergism for the mixture. The individual toxic units (TU) were 0.06, 0.05, 0.09, and 0.16 for As, Cd Hg, and Pb, respectively and the summed toxic unit (TU) was 0.37 (less than 1). This study provides needed scientific data necessary for carrying out complete risk assessment of As, Cd, Hg, and Pb mixtures of some priority compounds.

## Introduction

Virtually, an unlimited number of different mixtures of pollutants occur in the environment, and the number and concentration of chemicals in these mixtures are highly variable. Metal pollutants can be classified as either essential elements (important for life) or nonessential elements (with no known physiological functions to humans). Non-essential metals (NEMs) are priority pollutants that pose potential risks to human health and the environment.

Exposure to NEMs such as arsenic, cadmium, lead and mercury has been associated with a significant number of adverse health effects in humans. Although most research with NEMs has been done with single or individual metals, in reality, organisms are often exposed to multiple contaminants at the same time through the air, food and water. One of the reasons for the lack of mixture studies is cost hence; there is a strong interest to simplify toxicity assays.

The microtox assay is not only fast and reliable, but, it can be easily modified for mixture analysis. The microtox assay is simple in that it assesses chemical toxicity by measuring the magnitude of chemical interference with normal biological function in the bioluminescence bacteria *Vibrio fischeri* [1]. The bacteria produce a visible light commonly used as a bioassay for environmental quality since bioluminescence, the biological production of visible light, has an ecological function [2].

In the test, a reduction of bioluminescence of the bacteria is a function of toxicity; hence the exposed concentration should not be lethal to the test organism. We tested the toxicity of four NEMs (arsenic, As; cadmium, Cd; mercury, Hg; and lead, Pb) individually and as a composite mixture using the microtox bioassay. The concentrations of each chemical in the mixture were based on multiples of their maximum contaminant levels (MCLs) set by the U.S. EPA. The set MCLs are 10, 5, 2 and 15 ppb for As, Cd, Hg and Pd respectively [3]. This study provides needed scientific data necessary for carrying out complete risk assessment of mixtures of some priority compounds

## Materials and Methods

### Reagents and Chemicals

Lyophilized/freeze dried Microtox reagent (*Vibrio fischeri*) – Lot No. ACV014-2, Osmotic adjustment solution (OAS) - Lot No. OAS007, reconstitution solution – Lot No. RSN006Y, and diluent –Lot # DIL015L) were purchased from SDI, Newark DE.

### Instrument

The study was carried out with a Microtox Model 500 Toxicity Analyzer System (Azur Environmental Carlsbad, CA). This model is equipped with a 30-well incubator block, read and reagent wells, all of which are temperature controlled by an internal incubation unit. Operating temperatures were internally regulated and set at 5.5 ± 1°C for the reagent vials and 15 ± 0.5°C for both incubator block and read well. Light output was read from the digital display.

### Microtox Acute Toxicity Assay

Detailed protocol for the tests has been described by Azur Environmental [4]. The procedure measured the relative acute toxicity of each NEM and a composite mixture of all the metals, producing EC_50_ (concentration effecting 50% reduction of bioluminescence) data. Each test run was done in duplicates including two controls (without a metal) and eight sample dilutions of the chemicals. Tests were carried out on 45.00, 22.50, 11.25, 5.63, 2.81, 1.41, 0.70, and 0.35 % of the original concentration of 20 times the % MCL; which translated into 200, 100, 40 and 300 ng/mL (ppb) for As, Cd, Hg and Pb, respectively. The ratio for the mixture from these concentrations was 10:5:2:15 for As, Cd, Hg and Pb, respectively.

Osmotic adjustment solution (OAS) was added to each test to ensure that the final NaCl concentration in each test cuvette remained above 2.0%. The test samples and diluent controls were equilibrated to the required temperature of 15 ± 0.5°C. One milligram of lyophilized reagent (*Vibrio fischeri)* was hydrated in 1 mL reconstitution solution and 10 μl of the hydrated reagent were added to all the cuvettes. Then the light outputs were measured before and after the desired incubation time (50 mins). The diluent controls were run simultaneously and the experiment repeated twice.

### Quality Control

For quality control purposes, the sensitivity of *Vibrio fischeri* over storage time was tested using phenol, a positive control for the reagent. Data were then compared with the microtox quality assurance guidelines [4].

### Data Analysis

Results were calculated in terms of *Y*; i.e. the ratio of light lost during test time (t). A Dose- response relationship curve was constructed by plotting the percentages of reduction in bioluminescence correspondence to *Y* values against metal concentrations. The EC_50_s were determined as the concentrations corresponding to 50% decrease in light output (*Y* = 1.0). Supporting computer software microtox version 7.3 with a standard log- linear model was used to calculate the effective concentration EC_50_s.

## Results and Discussion

**Figure 1:**
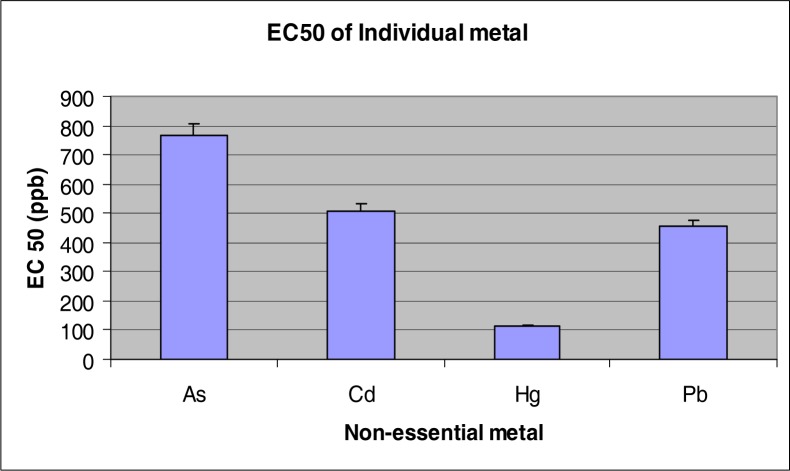
EC_50_ of metals tested individually

**Figure 2:**
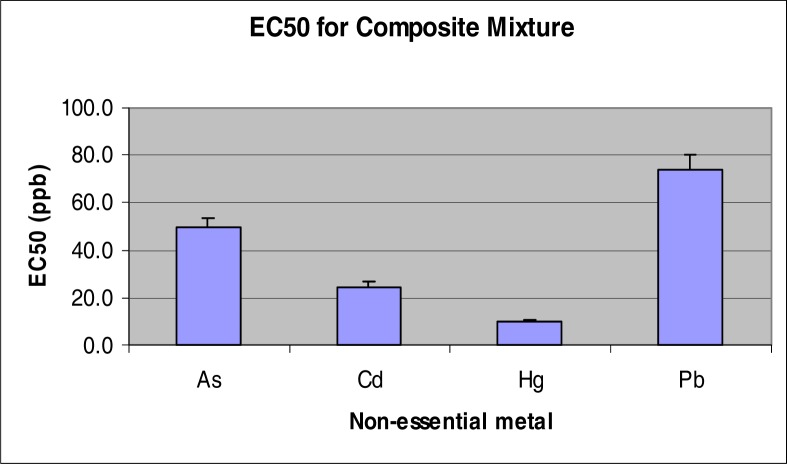
EC_50_ of individual metals in the composite mixture

Among the individual metals tested, the ranking of toxicity was Hg>Pb>Cd>As based on the EC_50_ values of 109, 455, 508 and 768 ppb for Hg, Pb, Cd and As, respectively (figure 1). The EC_50_ for the composite mixture was 495% MCL which translated into nominal concentrations of 49, 25, 10 and 74 ppb for As, Cd, Hg, and Pb, respectively (figure 2). Overall, the EC50 value of each NEM within the mixture was lower than the EC50 of the individual chemical; an evidence of synergism for the mixture.

The toxic unit (TU) approach was also used to evaluate the lethal mixture toxicity of the chemicals [5, 6]. TU is defined as the concentration of a chemical in a toxic mixture divided by its individual toxic concentration for the endpoint measured. Mixtures with summed TU values close to 1.0 are considered to be additive in toxicity, those with summed TU> 1.0 are less than additive in toxicity (antagonistic) and those with summed TU<1 are greater than additive in toxicity (synergistic). The individual TUs were 0.06, 0.05, 0.09 and 0.16 for As, Cd, Hg, and Pb, respectively (table 1) and the summed TU was 0.37 (less than 1) indicating synergistic effects. The ranking of the TUs was Pb>Hg> As> Cd which is different from the ranking made by Verslycke [7]. In their study with mysid *Neomysis integer* the ranking they observed was Hg> Pb>Cd. This difference could be attributed to the difference between the test organisms.

## Figures and Tables

**Table 1:** Toxic Unit (TU) calculated for each metal

*Metal*	*Toxic Unit*
As	0.064
Cd	0.049
Hg	0.090
Pb	0.163

*Sum*	*0.366*
